# Mitotic protein kinase-driven crosstalk of machineries for mitosis and metastasis

**DOI:** 10.1038/s12276-022-00750-y

**Published:** 2022-04-04

**Authors:** Chang-Hyeon Kim, Da-Eun Kim, Dae-Hoon Kim, Ga-Hong Min, Jung-Won Park, Yeo-Bin Kim, Chang K. Sung, Hyungshin Yim

**Affiliations:** 1 Department of Pharmacy, College of Pharmacy, Institute of Pharmaceutical Science and Technology, Hanyang University, Ansan, Gyeonggi-do 15588 Korea; 2 Department of Biological and Health Sciences, Texas A&M University-Kingsville, Kingsville, TX 78363 USA

**Keywords:** Oncogenes, Mitosis, Metastasis

## Abstract

Accumulating evidence indicates that mitotic protein kinases are involved in metastatic migration as well as tumorigenesis. Protein kinases and cytoskeletal proteins play a role in the efficient release of metastatic cells from a tumor mass in the tumor microenvironment, in addition to playing roles in mitosis. Mitotic protein kinases, including Polo-like kinase 1 (PLK1) and Aurora kinases, have been shown to be involved in metastasis in addition to cell proliferation and tumorigenesis, depending on the phosphorylation status and cellular context. Although the genetic programs underlying mitosis and metastasis are different, the same protein kinases and cytoskeletal proteins can participate in both mitosis and cell migration/invasion, resulting in migratory tumors. Cytoskeletal remodeling supports several cellular events, including cell division, movement, and migration. Thus, understanding the contributions of cytoskeletal proteins to the processes of cell division and metastatic motility is crucial for developing efficient therapeutic tools to treat cancer metastases. Here, we identify mitotic kinases that function in cancer metastasis as well as tumorigenesis. Several mitotic kinases, namely, PLK1, Aurora kinases, Rho-associated protein kinase 1, and integrin-linked kinase, are considered in this review, as an understanding of the shared machineries between mitosis and metastasis could be helpful for developing new strategies to treat cancer.

## Cancer: Linking cell division to cancer spread

Improving understanding of the mechanisms linking cell division and cancer spread (metastasis) could provide novel strategies for treatment. A group of enzymes involved in cell division (mitosis) are also thought to play critical roles in the spread of cancers. Hyungshin Yim at Hanyang University in Ansan, South Korea, and co-workers in Korea and the USA reviewed the roles of several mitotic enzymes that are connected with metastasis as well as tumorigenesis. They discussed how these enzymes modify cytoskeletal proteins and other substrates during cancer progression. Some regulatory control of cell cytoskeletal structures is required for cancer cells to metastasize. Recent research has uncovered crosstalk between mitotic enzymes and metastatic cytoskeletal molecules in various cancers. Targeting mitotic enzymes and the ways they influence cytoskeletal mechanisms could provide valuable therapeutic strategies for suppressing metastasis.

## Introduction

Emerging evidence indicates that mitotic protein kinases are involved in metastatic motility as well as tumorigenesis^1–9^. Previous studies have demonstrated mitotic rounding-up and cell migration crosstalk between adhesion complexes and the mitotic machinery^10–13^. In cancer cells, protein kinases and cytoskeletal proteins function in cell division as well as metastatic cell migration and invasion. Mitotic protein kinases have been shown to be involved in cancer metastasis in addition to cell proliferation and tumorigenesis^1–7^. For example, the mitotic kinase Polo-like kinase 1 (PLK1) induces epithelial-mesenchymal transition (EMT) in prostate cancer and non-small-cell lung cancer (NSCLC)^3,4,14^. It phosphorylates the intermediate filament protein vimentin during mitotic division and metastatic invasion, depending on the cellular context^3,15,16^. In addition, for mitotic rounding-up in prophase, mitotic cyclin-dependent kinase 1 (CDK1) phosphorylates Ect2, whose expression is elevated in several cancer types^13^. Ect2 facilitates metastasis and cell cycle progression by modulating the actin cytoskeleton^13^. Abnormal regulation of mitotic protein kinases can lead to metastatic invasion due to exploitation of the mitotic and cytoskeletal machineries for efficient release of metastatic cells from a tumor mass. A recent study supports the idea that chromosomal instability caused by defects in chromosomal segregation during mitosis induces cancer metastasis through secretion of genomic DNA into the cytosol, resulting in stimulation of NF-κB signaling and activation of an invasion-metastasis cascade^17^.

Knowledge of the cellular machinery involved in cancer tumorigenesis and metastasis is important for developing cancer therapies because the predominant cause of death in cancer patients is metastasis (which accounts for up to 90% of cancer deaths depending on the cancer type)^18^. For cell movement and shape alteration, the cytoskeleton must be reorganized both spatially and temporally^19^. Remodeling of the cytoskeleton is involved in several cellular events, including cell division, movement, and migration. Here, we review the role of cytoskeletal proteins in cell division and cell motility. More importantly, we explore which mitotic kinases function in cancer metastasis as well as tumorigenesis. In particular, we focus on PLK1, Aurora kinases, Rho-associated protein kinase 1 (ROCK1), and integrin-linked kinase (ILK) because these mitotic kinases regulate mitotic dynamics and metastasis via posttranslational modification of several substrates, including cytoskeletal proteins, in a manner dependent on extracellular signaling and the tumor microenvironment, as discussed below.

## Role of the cytoskeleton in cell division and metastatic motility

### Cell division

**Fig. 1:**
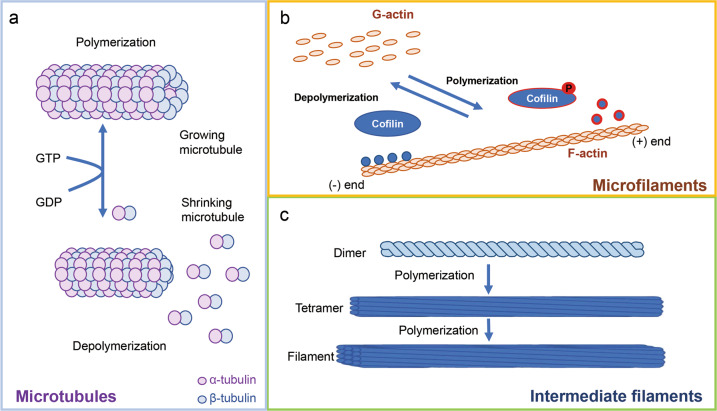
Three types of cytoskeletal filaments. **a** Polymerization and depolymerization of microtubules through GDP/GTP exchange of α- and β-tubulin heterodimers. **b** Polymerization and depolymerization of microfilaments through phosphorylation of cofilin. G-actin, monomeric actin form; F-actin, polymeric actin form. **c** Assembly of intermediate filaments (e.g., vimentin, desmin, laminin, keratin, and neurofilaments) via polymer formation.

**Fig. 2:**
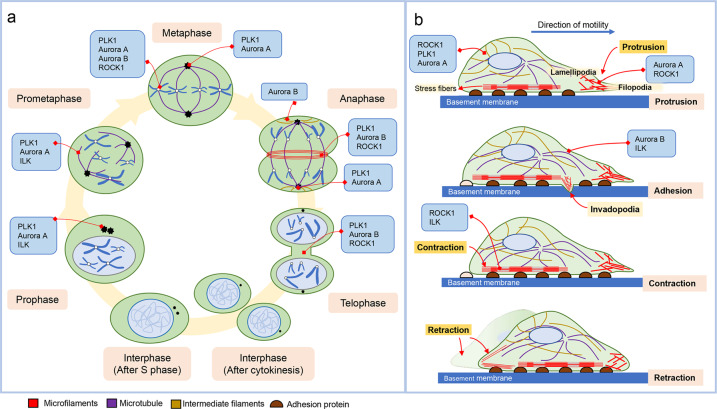
Locations of cytoskeletal filaments and mitotic protein kinases during cell division and cell migration. **a** During cell division, the mitotic kinases Aurora A/B, PLK1, ROCK1, and ILK are found in several locations, such as the kinetochore, spindle pole, or central spindle, where cytoskeletal filaments are present for mitotic dynamics. **b** The locations of cytoskeletal filaments and mitotic kinases (Aurora A/B, PLK1, ROCK1, and ILK) during the four processes of cell migration: protrusion (lamellipodia and filopodia), adhesion, contraction, and retraction.

**Fig. 3:**
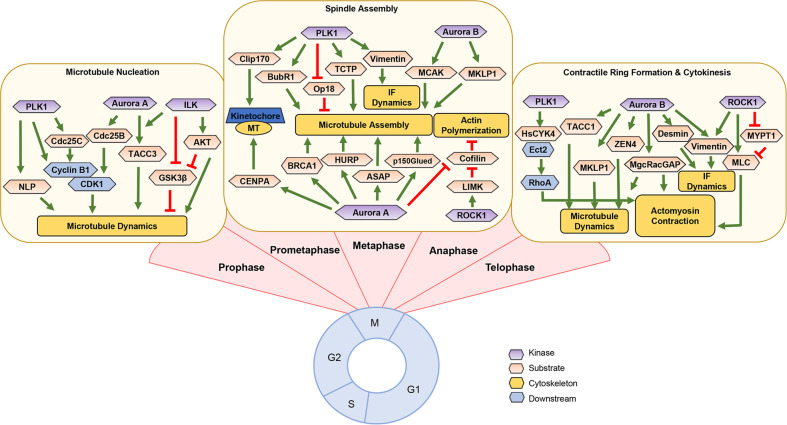
Phosphorylation of cytoskeletal filaments and/or related molecules by mitotic protein kinases during cell division. During cell division, the mitotic kinases Aurora A/B, PLK1, ROCK1, and ILK interact with and phosphorylate several mitotic substrates regulating cytoskeletal filaments for microtubule nucleation in prophase, spindle assembly in prometaphase and metaphase, and contractile ring formation in late mitosis.

Cytoskeletal filaments are important in determining cell shape, dynamic mitotic division, cell movement, intracellular transport, and metastasis^19,20^. There are three types of cytoskeletal filaments: microfilaments (e.g., actin), microtubules (e.g., tubulin), and intermediate filaments (e.g., vimentin, desmin, laminin, keratin, and neurofilaments), all of which are involved in cellular events, including cell division and cell movement through polymerization and depolymerization processes^19,21^ (Fig. 1). In the early stage of mitosis, tubules interact to form a microtubule seed for microtubule nucleation, resulting in growth of microtubules composed of spindle fibers^21,22^. When spindle fibers appear, chromosomes condense and attach to the microtubules of bipolar spindles and are pulled apart as sister chromatids to opposite poles of the cell^23^. Bipolar mitotic spindles are organized by centrosomes in the majority of cells, which are the principal microtubule-organizing centers (MTOCs) and regulate microtubule organization and γ-tubulin ring complexes (γ-TuRC). During mitosis, centrosomes nucleate an array of microtubules that are more dynamic than their corresponding interphase counterparts^21,23^. In addition, the actin cytoskeleton undergoes dramatic alterations and remodeling for cell division, resulting in the typical round shape of mitotic cells. The actin network is involved in centrosome separation and is dependent on cortical actin and myosin. At the cleavage furrow, actin rearranges and forms a contractile ring for cytokinesis (Fig. 2a). Phosphorylation of myosin light chain (MLC) regulates actomyosin contractility, allowing interaction between myosin and actin filaments to produce a mechanical force in cytokinesis^24,25^ (Fig. 3). The cleavage furrow is primarily regulated by a complex of actin and myosin filaments. The small GTPase Rho localizes at the cleavage furrow as a source of energy, and coordinated exchanges between the active GTP-bound and inactive GDP-bound forms of this protein are crucial for completion of cytokinesis^26,27^. Ect2, one of the guanine nucleotide-exchange factors that activate Rho family proteins, is required for accumulation and activation of GTP-RhoA in the cleavage furrow during cytokinesis^28^. Therefore, cytoskeletal molecules such as microtubules, actin, and myosin are central factors that contribute to dynamic morphological alterations and shape changes during mitotic progression.

### Cytoskeleton in cancer metastatic motility and invasion

The migration and invasion of cancer cells during metastasis are triggered by chemokines and growth factors, including *transforming growth factor*-β *(*TGF-β), epidermal growth factor (EGF), and Wnt, and involve the loss of cell–cell adhesion through EMT caused by changes in gene expression and posttranslational modifications^29,30^. Genetic alterations and posttranslational modification regulate factors involved in cell polarity, cell motility and extracellular matrix degradation during the early stages of metastasis^30,31^. During metastasis, cytoskeletal machineries regulate cell movement, resulting in the dissemination of cancer cells^19^. Major molecular factors involved in cell migration are small GTPases, such as Rho, Rac, and Cdc42, which are involved in cytoskeleton remodeling; Arp2/3, which is involved in actin remodeling; and myosin family proteins, which play a role in cell protrusion and contraction^32^ (Fig. 2b). Migration of cells involves four steps: protrusion (lamellipodia and filopodia), adhesion, contraction, and retraction^11,33^. In response to EMT-inducing factors such as TGF-β and EGF, the following cellular events occur sequentially, resulting in migration: actin polymerization, protrusion, adhesion linking, actomyosin contraction, disassembly of adhesion at the rear of the cell, and retraction of the trailing cell body^19,33^. As protrusion is induced by Rac/Cdc42-regulated actin polymerization, polymeric actin filaments (F-actin) are important in mesenchymal cell motility. Adhesion results in stabilization of protrusions. In addition, Rho family GTPases are critical for F-actin and focal adhesion organization^19,33^. During adhesion, several structural and regulatory proteins are recruited to the complex. Actomyosin, a contractile complex of F-actin and myosin, mediates cell spreading and adhesion to the extracellular matrix^19,33^. F-actin lengthens from focal adhesions at the leading edge of cell protrusions, lamellipodia, and filopodia (Fig. 2b). Myosin-driven F-actin merging is assumed to pull on strong focal adhesions at the front during migration and pull on weaker focal adhesions at the rear, moving the cell body forward^11,33^ (Fig. 2b). These adhesions then have to dissemble as the rear retracts. The high tension exerted on the rear adhesions contributes to detachment of the cell. For cells to metastasize, regulation of the cytoskeletal structure is required.

In summary, cytoskeletal proteins change cell shape, are involved in chromosomal segregation, and produce new cells during cell division. In cancer metastasis, they regulate cell movement to other regions and loss of cell–cell adhesion depending on extracellular signaling in the tumor microenvironment.

## PLK1 in tumor progression and metastasis

### Mitotic function of PLK1

PLK1 is a master mitotic kinase in cell division, being involved from centrosomal maturation to mitotic exit^28,34–39^. PLK1 expression and activity increase from the G2 phase and peak in mitosis, and their location dramatically transitions from the centrosome and spindle pole to the central spindle, suggesting that several factors interact with PLK1^34,35,37^. PLK1 has a catalytic ATP-binding domain at the amino terminus and two substrate-binding polo-box domains at the carboxyl terminus^34,35^. For mitotic entry, PLK1 binds to and phosphorylates several mitotic substrates, such as cyclin B1 and Cdc25C, resulting in the activation of mitotic CDK1 for mitotic entry^34,35^ (Fig. 3). Phosphorylation of nine-like protein (NLP) induces cells to switch from G2 phase to mitotic phase as a result of NLP release from the centrosome for centrosomal maturation and microtubule nucleation^22^. PLK1 also regulates bipolar spindle formation by phosphorylating BubR1 and PICH1^35,36^.

The microtubule-stabilizing protein translationally controlled tumor protein (TCTP) and the microtubule-destabilizing oncoprotein Op18 are also regulated by PLK1^40,41^. TCTP is recruited to the mitotic spindle during metaphase by binding microtubules and released at the end of mitosis^41^. For the formation of kinetochore-microtubule attachments, Clip170 is phosphorylated by PLK1 and CK2 at Ser195 and Ser1318, respectively, for the interaction and recruitment of dynactin to the microtubule (+) ends and linkage of microtubules to the cortex through Cdc42 and IQGAP^39,42^ (Table 1). However, phosphorylation of Op18 by PLK1 at Ser16, Ser25, and Ser39 in *Xenopus* negatively regulates microtubule stabilization through inactivation of Op18, suggesting that PLK1 promotes microtubule stabilization by inhibiting Op18^40^. In addition, PLK1 regulates the microtubule depolymerase mitotic centromere-associated kinesin (MCAK), a key regulator of mitotic spindle assembly and dynamics, for chromosomal segregation^43^. PLK1-mediated MCAK phosphorylation promotes microtubule depolymerase activity, which is essential for faithful chromosome segregation, in addition to regulating microtubule dynamics and chromosome stability during cell division^43^. HsCYK4 and Ect2 are essential for stimulating RhoA-dependent contractile ring assembly at anaphase onset^38^. PLK1-mediated priming phosphorylation of HsCYK4, a subunit of the centralspindlin complex, functions in targeting Ect2 to the spindle midzone and activating the Ect2-RhoA network to induce formation of the cleavage furrow^38^. During late telophase of mitosis, the intermediate filament vimentin functions in cytokinesis. During mitosis, vimentin is phosphorylated by PLK1 and CDK1 and loses its filament-forming ability^16^. Thus, phosphorylation of mitotic factors by PLK1 is important in the regulation of the cytoskeletal network during mitosis.

**Table 1:** Substrates of PLK1 involved in cytoskeletal regulation in mitosis and metastasis.

Mitosis			
Substrate	Phosphorylation sites	Mitotic function	Reference
NLP	Thr22, Ser87, Ser88, Thr161, Ser349, Ser498, Ser670, Ser686	Centrosomal maturation	^22^
BubR1	Ser676	Bipolar spindle formation	^36^
TCTP	Ser46, Ser64	Microtubule dynamics	^41^
CLIP170	Ser195	Kinetochore-microtubule attachment	^39^
Op18	Ser16, Ser25, Ser39	Microtubule stabilization	^40^
MCAK	Ser592, Ser595, Ser621, Ser633, Ser715	Chromosome segregation	^43^
HsCYK4	Ser157	Chromosome segregation	^38^
Vimentin	Ser82	Intermediate filament dynamics for cytokinesis	^16^

Metastasis			
Substrate	Phosphorylation sites	Metastatic function	Reference
c-Raf	Ser338, Ser339	MEK1/2-ERK1/2-Fra1-ZEB1/2 signal activation	^4^
Vimentin	Ser83, Thr327, Ser339	Disassembly from filament bundles, Smad translocation, and PD-L1 and Slug expression	^3,15^

### Clinical relevance of PLK1 in cancer

Because PLK1 plays a major role in cell division and its transient overexpression induces a transformed phenotype in murine fibroblasts^44^, it has been suggested to function as an oncogene when overexpressed. A recent study revealed that Plk1 overexpression in mouse embryo fibroblasts promoted oncogenic transformation as determined by colony formation in soft agar after 4 weeks. In addition, *Plk1* transgenic mice that express Plk1 in a graded manner had a shorter lifespan than wild-type mice due to increased tumor incidence^45^. The expression of PLK1 is high in diverse leukemias and carcinomas, including NSCLC, head and neck, esophageal, pharynx, liver, breast, colon, ovarian, stomach, pancreatic, and prostate cancers, and melanoma^34,46–50^. Clinical studies also revealed that a high level of PLK1 was correlated with low survival of patients with several types of carcinoma, including NSCLC^49^, head and neck cancer^46^, and esophageal cancer^51^. Among 121 NSCLC patients, those with high *PLK1* expression had a 5-year survival rate of 24.2%; in contrast, those with moderate expression of *PLK1* had a 5-year survival rate of 51.8% (*P* = 0.001)^49^, indicating that *PLK1* expression is a strong negative prognostic indicator, at least in NSCLC. In a study of 49 esophageal cancer patients, the 3-year survival rate was 54.9% in patients with low *PLK1* expression but 24.8% in those with high *PLK1* expression (*P* < 0.05)^51^. Based on these reports, the PLK1 expression level is a useful prognostic marker in several carcinomas.

In addition, studies of PLK1 expression and tumor stage have revealed that its expression is positively correlated with the clinical stage of NSCLC^14^, colon cancer^47^, prostate cancer^48^, endometrial cancer^52^, breast cancer^50^, and ovarian cancer^53^. In patients with endometrial cancer, the PLK1-stained cell population increased with tumor grade^52^. In ovarian cancer patients, the PLK1-positive cell population also increased with cancer stage: 14.74 ± 5.81%, 23.00 ± 9.610%, and 32.55 ± 9.36% in stages 1, 2, and 3, respectively^53^. The PLK1-positive cell population was significantly higher in stage 3 ovarian cancer than in stage 1 ovarian cancer (*P* = 0.001). These studies concluded that PLK1 expression is related to malignancy and a high risk of cancer metastasis. A meta-analysis of breast cancer patients revealed that high PLK1 expression was correlated with large tumor size (>2 cm) (*P* < 0.001) in 1779 breast cancer patients, higher tumor grade (*P* < 0.001) in 1989 patients and, lymph node metastasis (*P* = 0.001) in 1975 patients^50^, suggesting that high PLK1 expression indicates poor clinical outcomes in breast cancer patients. This phenomenon is not limited to breast cancer. Significant positive correlations were found between PLK1 expression and histologic grade (*P* = 0.005), recurrence (*P* < 0.001), and metastasis (*P* = 0.001) in urothelial carcinoma of the bladder, suggesting that PLK1 expression status is closely correlated with important histopathologic characteristics, including the grade and stage of bladder urothelial carcinoma^54^. In addition, PLK1 expression is correlated with the recurrence and metastasis of bladder carcinomas. Therefore, high expression of PLK1 is correlated with clinical outcomes in several cancers, including metastatic cancers.

### PLK1-driven metastasis

**Fig. 4:**
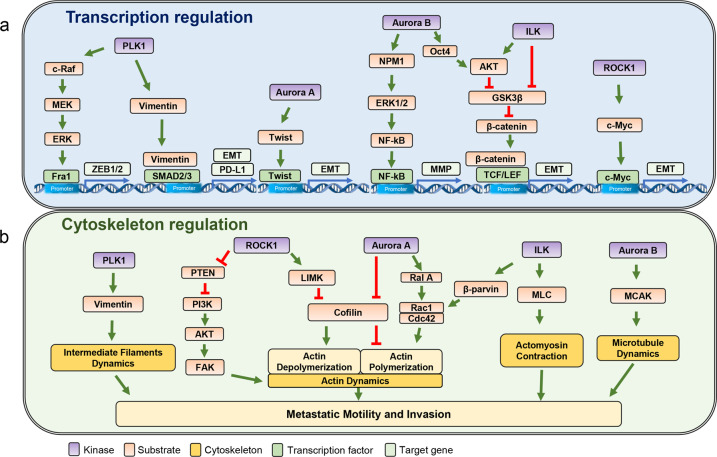
Regulation of cytoskeletal filaments and/or related molecules by mitotic protein kinases during metastasis. **a** Transcriptional and **b** cytoskeletal regulation by mitotic protein kinases through phosphorylation during cell migration and invasion of metastatic cancer cells.

Given that PLK1 expression is elevated in metastases of several cancer types, it is plausible that PLK1 can affect cancer metastasis. PLK1 expression was found to induce EMT in prostate epithelial cells via oncogenic transformation and transcriptional reprograming^4^. According to that study, inhibition of PLK1 in metastatic prostate cancer cells triggered epithelial characteristics and suppressed cell migration. PLK1-induced phosphorylation of c-Raf at Ser338 and Ser339 was suggested to contribute to c-Raf autophosphorylation of Ser621 as a mechanism of tumorigenesis and metastasis^4^. PLK1-dependent c-Raf phosphorylation induces stimulation of MEK1/2-ERK1/2-Fra1-ZEB1/2 signaling^4^ (Fig. 4).

Our recent study demonstrated that PLK1 itself drives cancer metastasis^14^. Since *PLK1* expression is a strong negative indicator of prognosis in NSCLC and is higher in metastatic NSCLC, we studied whether active PLK1 drives cancer metastasis^14^. Interestingly, PLK1 phosphorylated at Thr210 (activated PLK1) is abundant in NSCLC during TGF-β-induced EMT. The presence of constitutively active PLK1 induced NSCLC metastasis in a tail-vein injection mouse model, and the specific PLK1 inhibitor volasertib inhibited NSCLC metastasis in this in vivo model^14^. PLK1 with intact ATP-binding and polo-box domains facilitated cell motility and invasion by activating EMT reprogramming and upregulating genes related to the TGF-β-related signaling pathway, amplifying the metastatic properties of NSCLC cells^14^. In addition, PLK1-mediated phosphorylation of the cytoskeletal molecule vimentin at Ser83, Thr327, and Ser339 resulted in the loss of filament-forming ability for cell migration^3,15^. The PI3K/AKT, MAPK, Ras, JAK/STAT, TLR, and TGF-β signaling pathways were found to be activated in NSCLC cells expressing phosphomimetic vimentin via KEGG pathway analysis of microarray data. Phosphorylated vimentin recruits p-Smad2/3 for nuclear localization, resulting in PD-L1 expression for immune suppression in NSCLC (Fig. 4a). The clinical correlation between the expression of both vimentin and PLK1 and the OS rate of LUAD patients was significant in lung adenocarcinoma^15^. Together, these studies indicate that elevated expression of PLK1 in metastatic NSCLC is correlated with the induction and regulation of metastasis.

## Aurora kinases in tumor progression and metastasis

### Mitotic function of Aurora A

As a member of the mitotic serine/threonine Aurora kinase family, Aurora A (also named STK15/BTAK) functions in centrosome maturation, accurate mitotic entry and bipolar spindle assembly^55^. This protein was originally called “Aurora” owing to the monopolar spindles induced by *AURORA* gene mutation, which are reminiscent of the North pole^56^. This protein has two lobes that align residues of the catalytic cleft. The A loop (residues 274–297) binds to the substrate^55^. For mitotic entry, Cdc25B is phosphorylated on Ser353 at the centrosome before chromatin condensation in late G2 phase, which triggers activation of CDK1 through dephosphorylation by Cdc25B and subsequent relocalization of cyclin B1/CDK1 to the nucleus for mitotic transition^57,58^. After breakdown of the nuclear envelope, TACC3 phosphorylation by Aurora A is required for centrosomal localization as it promotes microtubule nucleation and stimulates microtubule growth^59^ (Table 2). In addition, CENPA phosphorylation at Ser7 by Aurora A is required for proper microtubule attachment at kinetochores from late prophase to metaphase^60^. Aurora A organizes spindles through regulation of the actin cytoskeleton^61^. Aurora A-mediated phosphorylation of cofilin at Ser3 results in actin polymerization, which progresses dynamically from prophase to metaphase. Aurora A located at the spindle pole phosphorylates p150Glued at Ser19 for central spindle assembly^62^ and mitotic BRCA1 at Ser308 to ensure proper microtubule plus-end assembly, increasing the rate of microtubule assembly^63^ (Fig. 3). Aurora A also regulates the microtubule-binding activity of HURP for mitotic spindle assembly^64,65^. A unique human microtubule-associated protein (MAP) involved in spindle formation, ASter-associated protein (ASAP), is also regulated by Aurora A through phosphorylation at Ser625^66^. Aurora A is degraded during the metaphase-anaphase transition by APC/C. In summary, Aurora A is essential for mitotic entry and spindle pole assembly through microtubule assembly.

**Table 2:** Substrates of Aurora A and Aurora B involved in cytoskeletal regulation in mitosis and metastasis.

Mitosis			
Aurora kinase	Substrate (P-residues)	Mitotic function	Reference
Aurora A	Cdc25B (Ser353)	Activation of cyclin B1/CDK1 to trigger the G2/M transition for mitotic entry	^57,58^
	TACC3 (Ser34, Ser552, Ser558)	Microtubule stability, stimulation of microtubule growth	^59^
	CENPA (Ser7)	Microtubule attachment at kinetochores	^60^
	p150Glued (Ser19)	Central spindle assembly	^62^
	BRCA1 (Ser308)	Microtubule assembly	^63^
	HURP (Ser627, Ser 725, Ser757, Ser830)	HURP stability for microtubule-binding	^64,65^
	ASAP (Ser625)	Spindle formation	^66^
	Cofilin (Ser3)	Actin polymerization	^61^
Aurora B	MCAK (Ser192)	Spindle formation and chromosomal segregation	^87^
	ZEN4 (Ser680)	Central spindle assembly and completion of cytokinesis	^88^
	MKLP (Ser708)	Central spindle assembly and completion of cytokinesis	^88^
	TACC1 (N.D.)	Formation of the midbody and completion of cytokinesis	^86^
	Desmin (Ser11, Thr16, Ser59)	Disassembly of desmin intermediate filaments	^85^
	MgcRacGAP (N.D.)	Completion of cytokinesis	^84^
	Vimentin (Ser72)	Segregation of vimentin intermediate filaments	^89^

Metastasis			
Aurora kinase	Substrate (P-residues)	Metastatic function	Reference
Aurora A	Twist (Ser123, Thr148, Ser184)	Transcriptional activation for cell motility during EMT	^81^
	RalA (Ser194)	Regulation of actin dynamics and cell motility	^82^
Aurora B	Oct4 (Thr343)	Activation of AKT and inactivation of GSK3β for stabilization of Snail	^8^
	NPM1 (Ser125)	Acceleration of tumor initiation and metastasis	^7^
	MCAK (Ser192)	Cell motility and directional migration	^87^

### Clinical relevance of Aurora A in cancer and metastasis

Overexpression of Aurora A induces chromosome instability and oncogenic transformation. Abnormal overexpression of Aurora A occurs in the early pathological stages of human ovarian tumorigenesis^67^ and pancreatic cell transformation^68^. Overexpression of Aurora A and loss of p53 function can accompany centrosome amplification and aneuploidy^69^. Aurora A phosphorylates p53 at Ser215 within the DNA-binding domain to suppress the transactivation activity of p53, suggesting that Aurora A inhibits the transactivation activity of p53 through suppression of DNA binding by direct phosphorylation^69^. In a conditional Aurora A expression system, Aurora A expression and p53 inactivation were required for tumorigenesis, which is supported by clinical observations in hepatocellular carcinoma^70^, indicating that overexpression of Aurora A and inactivation of p53 contribute cooperatively to tumor formation.

Clinically, overexpression of Aurora A is strongly associated with the low survival rates of diverse carcinomas including breast^71^, ovarian^72^, gastric^73^, colorectal^74^, and non-small-cell lung carcinoma^75^. In 151 colorectal adenocarcinoma patients, Aurora A was overexpressed as determined by immunohistochemical analysis of tissue microarrays^74^. Expression of Aurora A was observed in 45% of patients (68/151). Progression-free survival was shorter in patients who expressed high levels of Aurora A than in those who expressed low levels of this protein (*P* < 0.001). Elevated expression of Aurora A was strongly associated with recurrence in stage 2 or 3 colon cancer, and a high level of Aurora A was associated with poor survival rates in colorectal cancer patients with liver metastasis. In addition, the prognostic significance of *AURKA* was assessed in breast cancer patients. Increased *AURKA* expression was strongly associated with poor survival rates (*P* < 0.05) based on KM-plotter analysis^76^. In ovarian cancer, the Aurora A level was correlated with the OS of patients with stage III ovarian cancer (*n* = 101) when its expression was stratified as none or marginal (score 0 + 1), low (score 2), or high (score 3)^72^.

In NSCLC patients, elevated Aurora A expression is associated with poor OS and clinical lymph node metastasis (*P* = 0.038)^75^. In addition, the *AURKA* level in tumor specimens was found to vary according to the status of differentiation; *AURKA* was highly expressed in poorly or moderately differentiated (grade 3 or 2) lung cancer specimens compared to well-differentiated cases (grade 1) (*P* < 0.01)^77^. The results of these studies indicate that Aurora A is a crucial factor in tumorigenesis and metastasis.

### Aurora A in cancer metastasis

Aurora A has been studied in several cancers including carcinoma of the breast^5,78^, esophagus^79^, and colorectum^80^ and NSCLC^75^. In esophageal cancer, Aurora A overexpression increased esophageal cancer cell invasion and migration owing to induction of matrix metalloproteinases (MMPs) as determined by treatment with an MMP inhibitor^79^, suggesting that Aurora A-induced cell invasiveness is dependent on MMPs. Similarly, in estrogen receptor α-positive breast cancer, overexpression of Aurora A resulted in EMT and a mesenchymal-like phenotype accompanied by high levels of CD44 and HER-2/Neu^5^. Aurora A was significantly elevated in metastatic colorectal cancer, which promoted slug activation and enhanced the metastatic capacity of colorectal cancer cells in vitro and in vivo^80^. The mesenchymal transcription factor Twist1 has been identified as a substrate of Aurora A for EMT in pancreatic cancer^81^. Phosphorylation results in stabilization of Aurora A and promotes EMT. Moreover, Aurora A phosphorylates substrates involved in cell movement such as the GTPase RalA^78,82^. Phosphorylation of RalA at Ser194 induces its activation and thus collagen-dependent cell motility and anchorage-independent growth in epithelial cells, although not in cancer cells^82^. This phosphorylation facilitates the association of RalA with RalBP1 for regulation of Cdc42 and Rac1, affecting actin dynamics^68^. In lymphatic metastatic breast cancer, Aurora A also upregulates nonphosphorylated, active cofilin by increasing the phosphatase SSH1 on the basis of immunohistochemistry analysis of 99 breast cancer tissues^78^. Aurora A enriched cofilin and actin reorganization, and Aurora A was shown to regulate cofilin activity by reducing phosphorylation^78,83^. Inhibition of Aurora A activity using specific shRNAs in a xenograft model suppressed the mesenchymal transition and restored an epithelial phenotype, consequently inhibiting distant metastatic processes^5^, suggesting that Aurora A is important for EMT and metastasis. The loss-of-function experiments described above suggest that inhibition of Aurora A is important in reducing the transition from epithelial to mesenchymal traits.

## Aurora B in tumor progression and metastasis

### Mitotic function of Aurora B

Aurora B functions as a mitotic protein kinase in the late stages of cell division, including processes such as spindle pole formation, chromosomal segregation, and cytokinesis^84–88^. Aurora B regulates the function of the kinesin-related protein MCAK, a microtubule depolymerase, at microtubule ends by phosphorylating Ser192 during metaphase, resulting in spindle formation and chromosomal movement^87^ (Table 2). ZEN4/MKLP1 is important for central spindle assembly and completion of cytokinesis, which is regulated by Aurora B^88^. For midbody formation and completion of cytokinesis, a TACC1–Aurora B complex forms and functions at the midbody for correct localization of the midzone spindle in late mitosis^86^. For completion of cytokinesis, the GTPase-activating proteins MgcRacGAP and Aurora B associate with each other^84^. Vimentin is phosphorylated by Aurora B at Ser72, contributing to intermediate filament formation at the bridge and to regulation of cleavage furrow formation during the transition from anaphase to cytokinesis^89^. Another intermediate filament, desmin, is distribution in the cytoplasm after phosphorylation by Aurora B^85^, which induces disassembly of filaments. Aurora B regulates the cleavage furrow and segregation of intermediate filaments coordinately with ROCK1 during cytokinesis^85^ (Fig. 3). Therefore, Aurora B functions from metaphase to cytokinesis by phosphorylating diverse cytoskeletal proteins and is a crucial mitotic kinase required for proper cell division.

### Clinical relevance of Aurora B expression in cancer

Accumulating evidence suggests that aberrant overexpression of Aurora B is associated with carcinogenesis. Increased levels of Aurora B have been observed in carcinomas of the breast^90^, lung^91^, stomach^92^, liver^93^, ovary^94^, prostate^95^, colon^96^, and thyroid^97^, as well as osteosarcomas^7^ and glioblastoma^98^. The correlation between Aurora B overexpression and poor prognosis (reduced OS and/or disease-free survival rates) has been reported for several cancers, including NSCLC, breast cancer and liver cancer^91,93,99^. In 312 breast cancer patients, higher levels of Aurora B were significantly correlated with poor survival (*P* = 0.038), indicating that elevated Aurora B contributes to a poor prognosis in breast cancer^90^.

In addition to the expression of Aurora B in diverse primary tumors, Aurora B is also expressed at high levels in advanced stages of cancer. In a clinicopathological analysis of 156 ovarian cancer patients, Aurora B expression was significantly higher in poor and moderately differentiated ovarian cancers (53.6% and 28.2% with high expression) than in well-differentiated cancers (10.0% with high expression) (*P* = 0.02). Another study demonstrated that over half of lymph node metastatic ovarian cancers highly expressed Aurora B (positive *vs*. negative expression, 50.8% *vs*. 27.3%) (*P* = 0.01)^94^. Aurora B expression was significantly higher in effusions than in primary tumors in advanced stages of ovarian cancer (*P* = 0.003), suggesting involvement of Aurora B in advanced cancer^100^. More directly, Aurora B expression was associated with lymph node metastasis in oral squamous cell carcinoma (OSCC). The average level of Aurora B-positive OSCC metastatic lymph nodes was higher than that in primary OSCC^6^. In addition, elevated expression of Aurora B is associated with early metastatic progression and aggressiveness of breast cancer^8^. In a breast cancer study, Aurora B expression was compared between normal tissues and cancer tissues using the TCGA database, and cancerous tissues were found to express significantly higher levels of Aurora B than normal tissues (*n* = 1215, *P* < 0.001). Therefore, Aurora B expression is correlated with the clinical stage of several tumor types.

### Function of Aurora B in cancer metastasis

Elevated expression of Aurora B is related to cancer metastasis based on the clinical correlation between its expression and the survival rate of cancer patients. There is evidence that Aurora B induces EMT in breast cancer, which depends on its kinase activity and stabilization of Snail^8^. Elevated Aurora B activates AKT by phosphorylating Oct4 at Thr343, resulting in activation of AKT and inactivation of GSK3β for stabilization of Snail, consequently inducing EMT and metastasis in breast cancer cells^8^ (Fig. 4a). Additionally, Aurora B-mediated phosphorylation of NPM1 at Ser125 accelerated tumor initiation and metastasis to pulmonary nodules in nude mice^7^. In that model, Aurora B promoted osteosarcoma metastasis through activation of the ERK/NF-κB/MMP axis as a result of NPM1 phosphorylation^7^. MCAK is phosphorylated by Aurora B at Ser192, which promotes endothelial cell polarity and directional migration. MCAK regulates directional migration by remodeling microtubules in the cytoskeleton, and phosphorylation plays an important role in this process^87^ (Fig. 4b). In contrast, depletion of Aurora B using RNA interference suppressed cell invasion and migration in cancers of the breast, stomach, and bone^8,92,101^. Therefore, upregulated expression and activity of Aurora B contribute to cancer cell invasion, migration, and metastasis in several cancer types, including breast cancer.

## ROCK1 in tumor progression and metastasis

### Function of ROCK1 in tumor progression

ROCK, a serine/threonine kinase, participates in cell movement by regulating the cytoskeleton^102,103^. Rho, a small GTPase, is activated by guanine nucleotide exchange factors (GEFs). Upon binding to GTP, RhoA activates ROCK, which regulates a wide range of cellular functions such as actin–myosin contraction, smooth muscle contraction, cell polarity, cell motility, and metastasis through phosphorylation of several substrates depending on the cellular context^27,102,104^. ROCK1 and ROCK2 share 65% sequence identity and contain an N-terminal kinase domain, a C-terminal pleckstrin homology domain, and cysteine-rich domains^104–106^. Both are expressed ubiquitously, while their subcellular localization, tissue-specific expression, and functions are different^107^. Interestingly, no cancer cells that lack both ROCK1 and ROCK2 have been found, indicating that ROCK proteins are essential for tumorigenesis^102^.

Functionally, ROCK1 activates LIMK1 via phosphorylation at Thr508, resulting in reorganization of the actin cytoskeleton^108^ (Table 3). During mitosis, LIMK1 regulates actin dynamics by phosphorylating and inactivating cofilin, an actin-depolymerizing protein, for accurate spindle orientation^108,109^. During cytokinesis, the association of Rho-GTP with ROCK1 activates ROCK1, which then phosphorylates several substrates, including vimentin, myosin-binding subunit of myosin phosphatase (MYPT1), and myosin light chain (MLC) (Fig. 3). ROCK1-mediated vimentin or MLC phosphorylation induces actomyosin contractility, allowing regulation of assembly-disassembly of vimentin at the cleavage furrow or the interaction between myosin II and actin filaments to produce mechanical force in cytokinesis, respectively^24,25,110^. ROCK1 also indirectly regulates MLC phosphorylation by phosphorylating and inactivating MYPT1^102,103^. Phosphorylation of the phosphatase MYPT1 at two inhibitory sites, Thr696 and Thr853, inactivates it, resulting in maintenance of MLC phosphorylation^111^.

**Table 3:** Substrates of ROCK1 involved in cytoskeletal regulation in mitosis and metastasis.

Mitosis			
Substrate	Phosphorylation sites	Mitotic function	Reference
LIMK1	Thr508	Phosphorylation of cofilin at Ser3 and its inactivation, resulting in stabilization of cortical actin networks	^108,109^
Vimentin	Ser71	Turnover of vimentin at the cleavage furrow	^110^
MLC	Thr18, Ser19	Actomyosin contraction	^24,25,102^
MYPT1	Thr696, Thr853	Actomyosin contraction	^111,140^

Metastasis			
Substrate	Phosphorylation sites	Metastatic function	Reference
MLC	Thr18, Ser19	Actomyosin contraction	^25,102^
LIMK1	Thr508	Inhibition of actin depolymerization	^2,108^
PTEN	N.D.	Activation of PI3K/AKT and polymerization of actin filaments for protrusion	^1,2^
c-Myc	Thr58, Ser62	Transcriptional stabilization and activation of c-Myc for metastatic regulation	^120,121^

### Clinical relevance of ROCK1 expression in cancer

Elevation of Rho and ROCK1 expression has been observed in several cancers, including colon carcinoma^112^, glioblastoma^113^, hepatocarcinoma^114^, melanoma^9^, papillary thyroid carcinoma^115^, and osteosarcoma^116^. Kaplan–Meier analysis revealed an inverse relationship between ROCK1 expression and survival in papillary thyroid carcinoma patients^115^. In addition, high expression of ROCK1 was associated with a more metastatic phenotype in bladder cancer^117,118^ and papillary thyroid carcinoma^115^. The RhoA and RhoC proteins and ROCK were more abundant in tumor tissue and metastatic lymph nodes than in normal bladder tissue and uninvolved lymph nodes (*P* < 0.0001)^117^. Moreover, elevated expression of ROCK1 was observed in late-stage (T2–T4) and large (≥ 3 cm) human urothelial bladder tumors compared with early-stage and small tumors, respectively^118^. These data indicate that ROCK1 is associated with the initiation and progression of bladder cancer. ROCK1 expression was markedly higher in PTC with lymphatic metastasis than in PTC without metastasis. In 225 cases of PTC (128 cases with lymphatic metastasis and 97 cases without lymphatic metastasis), qRT–PCR analysis revealed that *ROCK1* expression was significantly higher in tumor tissue than in normal adjacent tissue. Additionally, the expression of *ROCK1* was greater in PTC with lymphatic metastasis than in PTC without lymphatic metastasis^115^. In NSCLC tissues and adjacent normal tissues from 30 patients, ROCK1 expression was significantly higher in NSCLC than in normal tissues (*P* = 0.0035). ROCK1 expression was also positively correlated with tumor size (*P* = 0.0038), clinical stage (*P* = 1.17E-05), and lymph node metastasis in NSCLC (*P* = 0.0012)^1^. Inhibition of ROCK1 using siRNA in NSCLC suppressed cancer cell proliferation and migration through downregulation of cyclin D and cyclin E^119^. Based on these studies, ROCK1 can be regarded as a marker of a poor prognosis in cancer and a promising target for cancer treatment.

### Function of ROCK1 in cancer metastasis

Rho/ROCK signaling is involved in morphological alterations and the metastatic behavior of tumors^2,102,103,112^. Recent studies revealed that ROCK1 functions in cancer metastasis through the phosphorylation of several cytoskeletal proteins^1,9,117^. Briefly, active Rho binds to ROCK1, resulting in activation of MLC and actomyosin contraction. In addition, ROCK1 phosphorylates LIMK1 for metastatic migration as well as mitosis, consequently inhibiting actin depolymerization through cofilin-actin-depolymerizing proteins^108^. Focal adhesion kinase (FAK) is required for mechanosensing and cell motility and regulates focal adhesion dynamics in membrane protrusion^2^. Recent studies have reported that FAK participates in cell migration and invasion through the PI3K/AKT pathway, which is linked with ROCK1-mediated cell migration and invasion in NSCLC^1^. ROCK1 knockdown not only suppressed the migration and invasion of lung carcinoma A549 and NCI-H1299 cells but also inhibited the adhesion of NSCLC cells^1^.

In breast cancer, MCF-7 cells overexpressing ROCK1 metastasized to the hind limbs, liver, and bone^120^. When mice were treated with siRNA targeting ROCK, all mice remained healthy, indicating that ROCK can induce metastasis. Liu and colleagues hypothesized that ROCK-mediated phosphorylation of c-Myc at Thr58 and/or Ser62 stabilized the transcriptional activity of c-Myc based on their observation that c-Myc expression in metastatic breast cancer cells was significantly increased in vitro and in vivo^120^. An interaction between ROCK and c-Myc has also been demonstrated in prostate cancer tumorigenesis^121^. Thus, the interaction between ROCK1 and c-Myc can contribute to tumorigenesis and metastasis. Together, these studies indicate that ROCK1 contributes to metastatic motility and invasion as well as dynamic mitotic events through regulation of cytoskeletal molecules.

## ILK in tumor progression and metastasis

### Mitotic function of ILK

ILK, a highly conserved serine/threonine kinase, plays an essential role in cell adhesion, cell migration and cell shape changes by interacting with the cytoplasmic domain of β1 and β3 integrins^122^. Since its identification as a protein that interacts with integrin β1 and β3, ILK has been referred to as an integrin-linked kinase^122^. ILK is localized at the centrosome and microtubules, forming a complex with tubulin^123^. ILK helps organize mitotic spindle formation; loss of its activity causes mitotic spindle defects including abnormal mitotic spindles, chromosome abnormalities, and lagging chromatids through disruption of Aurora A/TACC3/ch-TOG interactions^10,124^. In addition, ILK facilitates the centrosomal localization of RUVBL1 and RUVBL2 via interaction and is involved in tubulin dynamics and mitotic spindle organization^10,125^. Furthermore, ILK phosphorylates AKT at Ser473 for activation, which affects AKT signaling^126^. Active AKT phosphorylates and inactivates GSK3β, an interphase regulator of microtubule dynamics at the centrosome, thereby affecting microtubule dynamics in the mitotic spindle^10,127^. ILK phosphorylates GSK3β on Ser9, which leads to its inactivation, indicating that ILK affects microtubule dynamics in mitosis by reducing the activity of GSK3β in both direct and indirect ways^127,128^. Therefore, ILK, as a centrosomal protein, regulates mitotic spindle organization for precise mitotic progression.

### Clinical relevance of ILK in cancer

ILK overexpression in epithelial cells induces tumorigenicity in nude mice, and ILK is recognized as a proto-oncogene^129^. Overexpression of ILK constitutively upregulated the levels of cyclin D and cyclin A in epithelial cells and consequently induced cell growth^129^. Suppression of ILK activity with specific inhibitors decreased tumor progression through G2/M phase arrest and apoptosis in glioblastoma cells^130^. Abnormal expression of ILK has been reported in diverse cancers, and it has been used as a biomarker for cancer diagnosis and prognostication^129,131,132^. The expression level of ILK and tumor grade were positively correlated in colorectal cancer^132^, breast cancer^131^, and NSCLC^129^. Evaluation of the transcript level of *ILK* in 64 breast cancer samples and immunohistochemical analysis of normal and cancerous tissues from 163 breast cancer patients revealed that the expression of ILK was significantly greater in cancer tissues than in normal adjacent tissues^131^. ILK expression was correlated with tumor size, grade, stage, and lymph node metastasis. In addition, the ILK level was highly correlated with OS based on Kaplan–Meier analysis^131^. Thus, ILK appears to be an important factor affecting the pathogenesis, progression, and prognosis of breast cancer. ILK expression was associated with NSCLC aggressiveness, with a positive correlation between preoperative adjuvant therapy and recurrence in NSCLC cells expressing ILK^129^. Immunohistochemical analysis of noncancerous pulmonary tissue and tumor tissues from NSCLC patients revealed that ILK was highly expressed in 31% of NSCLC tissues, while it was not detected in normal tissues. Expression levels were high in advanced T and N stage cancers^129^. These data indicate that ILK expression is correlated with the pathogenesis and progression of diverse cancers and that it is a diagnostic and prognostic marker for cancer.

### ILK in metastasis

Since ILK is a proto-oncogene and regulates cell motility, gain-of-function experiments were performed to determine whether it can drive EMT^132,133^. Ectopic overexpression of ILK in lung cells promoted cell invasiveness and had a significant impact on EMT, with increased expression of mesenchymal markers^132,133^. Immunohistochemical analyses of 108 primary tumor tissues derived from lung cancer patients revealed that ILK was highly expressed in 30.6% (33/108) of tumors. ILK expression was positively correlated with lymph node metastasis, indicating that ILK has a causal role in metastasis of lung cancer^133^ and colorectal cancer^132^. The level of ILK was correlated with tumor grade (*P* = 0.005), TNM stage (*P* < 0.001), and invasion depth (*P* < 0.001), suggesting that it is correlated with tumor progression^132^. Loss-of-function experiments using siRNA or shRNA targeting ILK have demonstrated that knockdown of ILK represses EMT, tumor growth, and metastasis in tongue and prostate cancers through inactivation of the PI3K/AKT pathway^134^.

ILK has several phosphorylation substrates, such as β-parvin, paxillin, GSK3β, and MLC, that function in the actin cytoskeleton^131^. ILK-mediated phosphorylation of β-parvin allows cells to spread and migrate through the activation of Rac1/Cdc42^135^. ILK phosphorylates GSK3β at Ser9 in breast cancer, affecting the activation of the transcription factor AP1 and the stabilization of β-catenin, which is indirectly related to deregulation of proliferation, migration, and differentiation^128,136^ (Table 4). ILK also directly phosphorylates MLC on Thr18 and Ser19 to promote cell contraction, motility, and migration^137–139^. ILK functions as an adapter protein through its kinase activity and regulates cell growth, EMT, invasion, migration, and tumor angiogenesis^122^. These data indicate that ILK, through phosphorylation of downstream proteins involved in cell movement and actin organization, is important for cancer metastasis as well as tumor progression.

**Table 4:** Substrates of ILK involved in cytoskeletal regulation in mitosis and metastasis.

Mitosis			
Substrate	Phosphorylation sites	Mitotic function	Reference
AKT	Ser473	Activation of AKT and inactivation of GSK3β for microtubule dynamics in the mitotic spindle	^10,126,127^
GSK3β	Ser9	Inactivation of GSK3β for microtubule dynamics in the mitotic spindle	^10,127,128^

Metastasis			
Substrate	Phosphorylation sites	Metastatic function	Reference
AKT	Ser473	Induction of angiogenesis and invasiveness	^141,142^
GSK3β	Ser9	Inactivation of GSK3β and stabilization of β-catenin for migration	^128,136^
MLC	Thr18, Ser19	Cell motility and migration	^137–139^

## Conclusions

Mitosis and metastasis are complex processes requiring dramatic reorganization of cytoskeletal molecules. Several mitotic protein kinases interact with cytoskeletal actin, myosin, and microtubule molecules or related factors directly or indirectly affecting cell shape and migration. Phosphorylation of cytoskeletal filaments regulates cell division, movement, and metastasis. Mounting evidence regarding crosstalk between mitotic protein kinases and metastatic cytoskeletal molecules raises the possibility that mitotic protein kinases function in cell migration during cancer metastasis (Tables 1–4). An understanding of the regulation of cellular machineries by mitotic protein kinases in metastasis could provide new strategies for cancer treatment. Importantly, there is clinical evidence that mitotic protein kinases are potential therapeutic targets for suppressing cancer metastasis as well as tumorigenesis. We anticipate that a deeper understanding of the cytoskeletal regulatory mechanisms that function in tumorigenesis by affecting cell division and that play role in cancer metastasis by affecting migration and invasion of cells will provide additional insight into the molecular basis of metastasis and potentially aid in the identification of new therapeutic targets.
